# Editorial: Plant bioactive compounds from agro-industrial by-products for improvement of nutritional quality of foods

**DOI:** 10.3389/fnut.2024.1448549

**Published:** 2024-06-28

**Authors:** Maria Celeiro, Ante Lončarić

**Affiliations:** ^1^CRETUS-Department of Analytical Chemistry, Nutrition and Food Science, University of Santiago de Compostela, Santiago de Compostela, Spain; ^2^Faculty of Food Technology, University of Osijek, Osijek, Croatia

**Keywords:** bioactive compound, agri-food by-product, functional food, green chemistry, polyphenols

The agro-industry generates a large amount of waste and by-products, which has a negative impact on the environment and the economy. Plant-based waste and by-products are rich in bioactive compounds such as polyphenols, dietary fiber, proteins, essential fatty acids, vitamin, and minerals, which can be exploited for improvement of nutritional Quality of Foods. Any part of plants, such as husks, seeds, leaves, roots, and stems, can be considered a source of bioactive compounds, and their valorisation is a suitable option for sustainability of food industry (1, 2).

There is a rising demand in maintaining human wellness through nutrition which has greatly promoted the interest in food bioactive compounds all over the world. Food bioactive compounds such as polyphenols, proteins, peptides, polysaccharides, phytochemicals, dietary fiber, and lipids can be extracted from agro-industrial by-products and wastes (3). Therefore, finding new or non-conventional sources, the new more “green” extraction methods, innovative approaches in designing functional foods and improving food properties has become a challenge for the food industry (4).

This Research Topic is aimed at collecting papers related to the obtention, characterization and application of plant-based bioactive compounds from agro-industrial by products. In this Research Topic there are four papers covering these aspects. They are briefly summarized below.

Zou et al. described the application, for the first time, of a cascade membrane technology to classify polysaccharides from the peels of stem lettuce, and three graded polysaccharides were obtained using ultrafiltration membranes in sequence. The physicochemical properties and immune-modulatory activity of three PPSLs fractions were analyzed and compared. Results showed that all three fractions have characteristic absorption peak of polysaccharides determined by FT-IR, and their monosaccharide composition only consisted of glucose determined by HPLC. The findings indicated that PPSL10 could be developed as immune-modulator in the field of functional foods.

Castillo et al. developed a scalable procedure with minimum energy requirements, Medium Scale Ambient Temperature (MSAT), in combination with solvents generally recognized as safe (GRAS), to obtain polyphenolic extracts from white grape (*Vitis vinifera*) marc. The operating parameters were optimized through a response surface matrix: extractor solvent volume, marc mass, and marc/dispersant mass ratio, using the total polyphenol content (TPC) and the antioxidant activity (AA) of the extracts as response parameters. In addition, the stability of the extracts was studied for 62 days. The effect of temperature, light exposure, and oxidative reactivity was evaluated. The bioactivity indices showed no changes with the storage conditions of the extracts in the first month of analysis, after which 75% of the antioxidant activity as the concentration of the polyphenolic profile remains. The absence of reactive oxygen and the cooling of the extract (4°C) were the most determining factors (*p* < 0.05) in modulating the stability of the total polyphenolic profile.

Moreno-Quiroga et al. evaluated the fruit mesocarps of *Cucurbita ficifolia* for physicochemical parameters, antioxidant activity, and phenolic compound contents in a collection of farmers' landraces. The results show that the content of soluble solid contents, pH, total sugars and flavonoids are influenced by the fruit's geographical origin (municipalities) and implicitly by their agroecological cultivation conditions among populations preserved by traditional farmers, significant differences and wide variability were found for all parameters evaluated. Eight compounds were identified by UPLC–MS, all with potential health effects. It was demonstrated that *C. ficifolia* fruit mesocarp has bioactive compounds with high antioxidant activity with the potential to both improve diet and to obtain other benefits against nontransmissible diseases derived from food and its associated risk factors.

Finally, a review was published by Regolo et al. showing how leaves can be exploited to manufacture many products in food (e.g., being incorporated in food formulations as natural antioxidants, or used to create edible coatings or films for food packaging), cosmetic and pharmaceutical industries (e.g., promising ingredients in anti-aging cosmetics such as oils, serums, dermatological creams, bath gels, and other products). This review focuses on the leaves' main bioactive compounds and their beneficial health effects, indicating their applications until today to enhance the leaves as a harvesting by-product and highlight their potential possible beneficial effects reuse for new potential healthy products.

In summary, the above-mentioned research works and reviews improve the knowledge of plant-based bioactive compounds from the agrifood sector to enhance the nutritional quality of foods or to obtain new products with added value.

## Author contributions

MC: Conceptualization, Writing – original draft. AL: Conceptualization, Writing – review & editing.
